# The HPV16 E6 Oncoprotein Causes Prolonged Receptor Protein Tyrosine Kinase Signaling and Enhances Internalization of Phosphorylated Receptor Species

**DOI:** 10.1371/journal.ppat.1003237

**Published:** 2013-03-14

**Authors:** Jennifer M. Spangle, Karl Munger

**Affiliations:** Division of Infectious Diseases, Brigham and Women's Hospital, Department of Medicine and Committee on Virology, Harvard Medical School, Boston, Massachusetts, United States of America; Indiana University, United States of America

## Abstract

The high-risk human papillomavirus (HPV) E6 proteins are consistently expressed in HPV-associated lesions and cancers. HPV16 E6 sustains the activity of the mTORC1 and mTORC2 signaling cascades under conditions of growth factor deprivation. Here we report that HPV16 E6 activated mTORC1 by enhanced signaling through receptor protein tyrosine kinases, including epidermal growth factor receptor and insulin receptor and insulin-like growth factor receptors. This is evidenced by sustained signaling through these receptors for several hours after growth factor withdrawal. HPV16 E6 increased the internalization of activated receptor species, and the signaling adaptor protein GRB2 was shown to be critical for HPV16 E6 mediated enhanced EGFR internalization and mTORC1 activation. As a consequence of receptor protein kinase mediated mTORC1 activation, HPV16 E6 expression increased cellular migration of primary human epithelial cells. This study identifies a previously unappreciated mechanism by which HPV E6 proteins perturb host-signaling pathways presumably to sustain protein synthesis during the viral life cycle that may also contribute to cellular transforming activities of high-risk HPV E6 proteins.

## Author Summary

High-risk human papillomavirus infections are associated with nearly all cases of cervical cancer. HPVs infect basal epithelial cells but virion production is restricted to the outer, terminally differentiated layers of the infected epithelia where supply of nutrients and growth factors may be limited. High-risk HPV E6 proteins have been shown to activate mTORC1 signaling and increase cap dependent translation. Here we show that HPV16 E6 activates the mTORC1 and MAP kinase signaling pathways through activating receptor protein tyrosine kinases (RPTKs) and increases EGFR internalization, even after growth factor withdrawal. The signaling adaptor protein GRB2 is a critical mediator of HPV16 E6 mediated EGFR internalization and mTORC1 activation. Lastly, we demonstrate that HPV16 E6 mediated activation of RPTK and mTORC1 signaling causes increased cellular migration even after growth factor withdrawal. These results suggest a previously unappreciated mechanism by which HPV E6 proteins may support the viral life cycle and that may contribute to the transforming activities of high-risk HPV E6 proteins. Hence, inhibition of RPTK signaling networks may be evaluated as a therapeutic strategy for HPV-associated lesions and cancers.

## Introduction

Human papillomaviruses (HPVs) are small viruses with double stranded DNA genomes that infect squamous epithelial tissue. Approximately 200 HPV types have been identified and categorized based on the type of host epithelial tissue they infect. A subset of HPVs infects the mucosal epithelium, and high-risk mucosal HPVs cause lesions that can undergo malignant progression. High-risk HPVs are the causative agents for nearly 100% of cervical cancers.

A frequent hallmark of HPV-associated carcinogenesis is the integration of the HPV genome into a host chromosome. This results in the dysregulated expression of the viral E6 and HPV E7 proteins. These two proteins together are sufficient to cause cervical carcinoma in a transgenic mouse model and are necessary for the maintenance of the transformed state of cervical cancer cell lines (reviewed in reference [1]). High-risk HPV E6 proteins form a complex with the E3 ubiquitin ligase UBE3A (E6AP) and the p53 tumor suppressor, targeting p53 proteasomal degradation [2]. The E6/UBE3A complex can also target other cellular proteins for degradation, including members of a diverse group of cellular PDZ proteins that associate with high-risk HPV E6 proteins through their carboxyl-terminal PDZ binding domains [3]–[10]. High-risk HPV E6 proteins also contribute to the immortalization of the host cell through transcriptional activation of hTERT, the catalytic component of the human telomerase enzyme [11].

HPVs infect the basal epithelial cells, which occupy a nutrient rich environment. These cells generally undergo asymmetric cell division, where one daughter cell remains a basal cell whereas the other daughter cell is poised to undergo differentiation to form the stratified squamous epithelium. Viral genome replication and progeny synthesis is confined to terminally differentiated cells in upper layers of the epithelium. Through inactivation of the p53 and pRB tumor suppressors, the HPV E6 and E7 proteins keep such cells in a DNA synthesis competent state but it is unknown how completion of the viral life cycle may occur in a cellular environment that presumably is restricted for nutrients and energy sources that are required for DNA replication and protein synthesis. HPV16 E7 expression has been reported to cause a metabolic switch from oxidative phosphorylation to anaerobic fermentation, a phenomenon referred to as the “Warburg effect” [12]. Moreover, HPV16 E7 expressing cells exhibit evidence of autophagy even when grown in rich growth media [13]. When deprived of serum, normal cells undergo G1 growth arrest, but HPV16 E7 expressing cells continue to proliferate and eventually expire through caspase independent cell death [14]. This reflects a cellular tumor suppressive response to HPV E7 expression that has been termed the trophic sentinel response [15]. Co-expression of HPV16 E6 abrogates HPV16 E7 triggered trophic sentinel signaling. Presumably to overcome the E7 triggered trophic sentinel response, HPV16 E6 activates mTORC1 signaling and increases protein synthesis under normal growth conditions and upon growth factor withdrawal [16]–[18].

HPV16 E6 activates mTORC1 through the upstream kinases PDK1 and mTORC2 under conditions of nutrient deprivation [16]. PDK1 is downstream of PI3K and is activated through several membrane-associated signaling events including the activation of ERBB, insulin receptor (INSR) and insulin-like growth factor receptor (IGFR) receptor protein tyrosine kinases (RPTKs). Following ligand binding, RPTKs dimerize, initiating receptor autophosphorylation on multiple tyrosine residues within the intracellular domains. Adaptor proteins are then recruited to the tyrosine phosphorylated receptors via their SH2 domains and activate downstream effector cascades. Activated EGFR associates with multiple signaling adaptor proteins including growth factor receptor-bound protein 2 (GRB2), Src homology 2 domain containing (SHC), and phosphoinositide phospholipase Cγ (PLCγ) [19]–[21]. Similarly, INSR and IGFR are autophosphorylated in a conserved kinase activation loop followed by tyrosine phosphorylation within the intracellular domain that triggers downstream effector signaling [22], [23]. ERBB and INSR/IGFR receptor activation trigger common downstream signaling cascades including AKT and mTORC1. ERBB and INSR/IGFR autophosphorylation induces receptor internalization through clathrin mediated endocytosis, an event that is associated with active signaling and can either lead to receptor recycling to the cell surface or receptor degradation [24], [25].

Here we report that HPV16 E6 expressing primary human foreskin keratinocytes (HFKs) show evidence of RPTK hyperactivity when grown in normal growth medium and that RPTK signaling is sustained over prolonged periods of time when the cells are deprived of the corresponding growth factors. We show that HPV16 E6 increases phosphorylation of INSR, IGFR, and EGFR RPTKs. We also show that HPV16 E6 expression causes an increase in the internalization of activated, phosphorylated RPTKs. We discovered that small molecule RPTK inhibitors as well as GRB2 depletion abrogates mTORC1 activity suggesting that E6 mediated mTORC1 activation is caused by enhanced RPTK activity. Moreover, we show that GRB2 depletion also specifically impairs EGF internalization in HPV16 E6 expressing cells. HPV16 E6 mediated RPTK activation causes an increase in mTORC1-dependent cellular migration in the absence of growth factors, which may be relevant to HPV associated carcinogenesis.

## Results

### Prolonged activity of receptor protein tyrosine kinase signaling pathways after EGF deprivation in HPV16 E6 expressing HFKs

**Figure 1:**
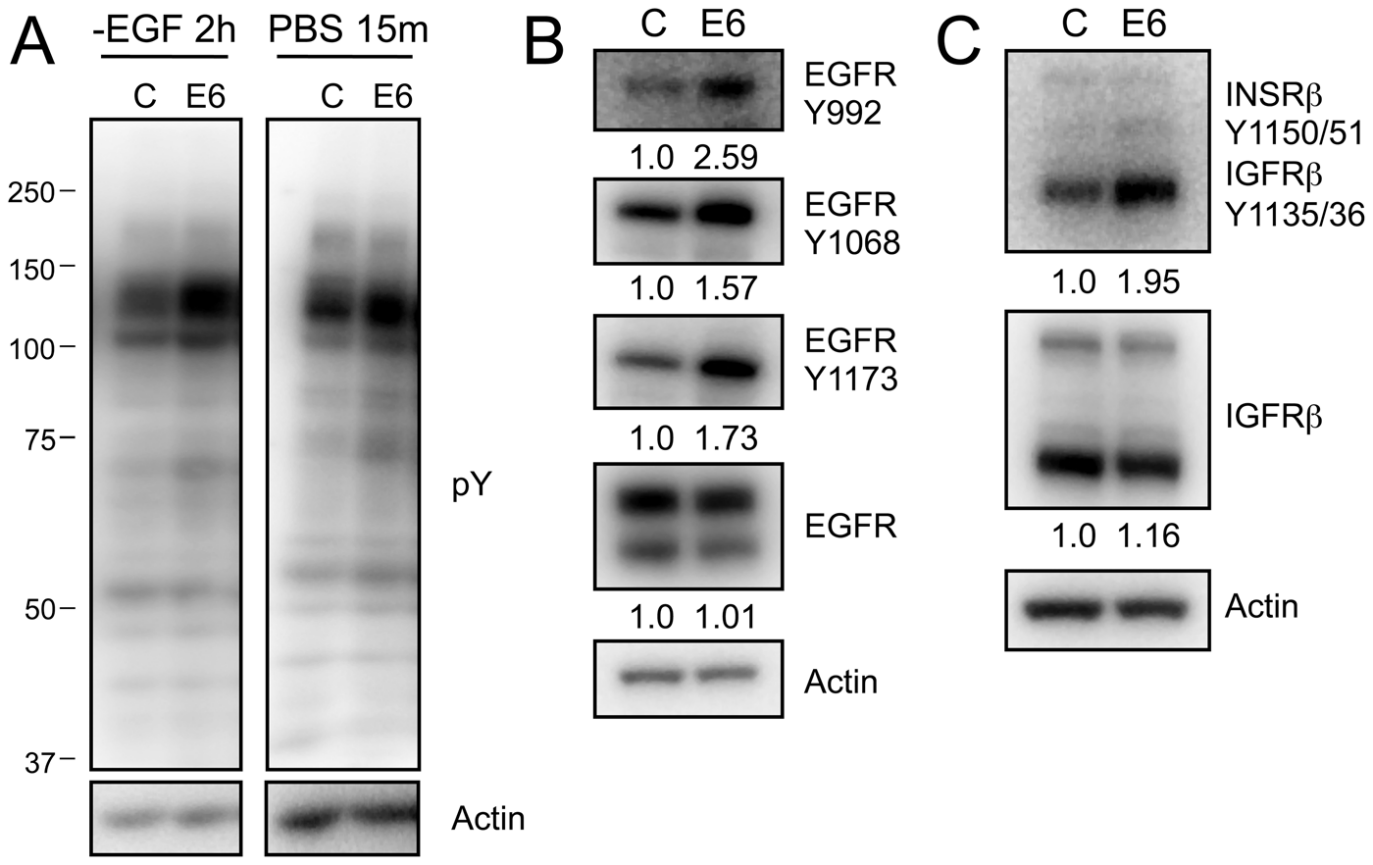
ERBB2 phosphorylation is increased in HPV16 E6 expressing HFKs. (**A**) Western blot analysis of proteins with phosphorylated tyrosine residues following EGF withdrawal (left) or PBS starvation (right) from HFK lysates stably expressing HPV16 E6 (E6) or the pLentiN6.3 control vector (C). (**B**) and (**C**) Western blot analyses probing the phosphorylation status of EGFR (**B**) or INSRβ/IGFRβ (**C**) using phosphospecific antibodies in HPV16 E6 (E6) or LXSN control vector (C) expressing stable HFKs. Representative experiments from three independent experiments are shown in panels A, B and C. Quantifications relative to actin are shown below each panel.

We previously reported that HPV16 E6 activates S6K and 4E-BP1 through mTORC1 and that persistent AKT phosphorylation under conditions of nutrient deprivation resulted from PDK1 and mTORC2 activation [16]. To determine the mechanism of activation, we first took a general approach and evaluated global tyrosine phosphorylation under conditions of growth factor deprivation (-EGF) and general nutrient deprivation (PBS). Activation of RPTK signaling is initiated by the autophosphorylation of tyrosine residues on the intracellular domains of these proteins. Hence, we evaluated global tyrosine phosphorylation in donor matched populations of primary human foreskin keratinocytes (HFKs) expressing HPV16 E6 or transduced with control vector by immunoblotting with a phosphotyrosine specific antibody. Compared to control cells, tyrosine phosphorylation was increased in HPV16 E6 expressing HFKs grown for 2 hours in the absence of EGF in the growth medium (Figure 1A, left) or under conditions of nutrient deprivation in PBS for 15 minutes (Figure 1A, right).

Autophosphorylation of specific tyrosine residues in the intracellular domain of EGFR causes recruitment of adaptor proteins and activation of effector signaling pathways. We therefore evaluated EGFR autophosphorylation at specific tyrosine residues. In EGF deprived HPV16 E6 expressing HFKs, we observed prolonged EGFR autophosphorylation on Y992, Y1068, and Y1173, which are associated with PLCγ activation, GRB2 binding and MAPK/PI3K signaling, and SHC binding and MAPK signaling, respectively (Figure 1B). To determine whether HPV16 E6 may be activating multiple RPTKs, we also evaluated the phosphorylation/activity status of insulin receptor-β (INSRβ) and insulin-like growth factor receptor-β (IGFRβ) utilizing an antibody that recognizes specific phosphorylated tyrosine resides on both receptors: Y1135/36 on INSRβ and Y1150/51 on IGFRβ. Similar to what we observed with EGFR, autophosphorylation of INSRβ and/or IGFRβ at these sites was sustained at higher levels in PBS treated HPV16 E6 expressing HFKs than in control HFKs (Figure 1C).

Thus activation of EGFR, and well as INSRβ and/or IGFRβ is sustained at higher levels and over prolonged periods of time in HPV16 E6 expressing HFKs after growth factor or nutrient deprivation.

### Activation of RPTK effector signaling in HPV16 E6 expressing HFKs

**Figure 2:**
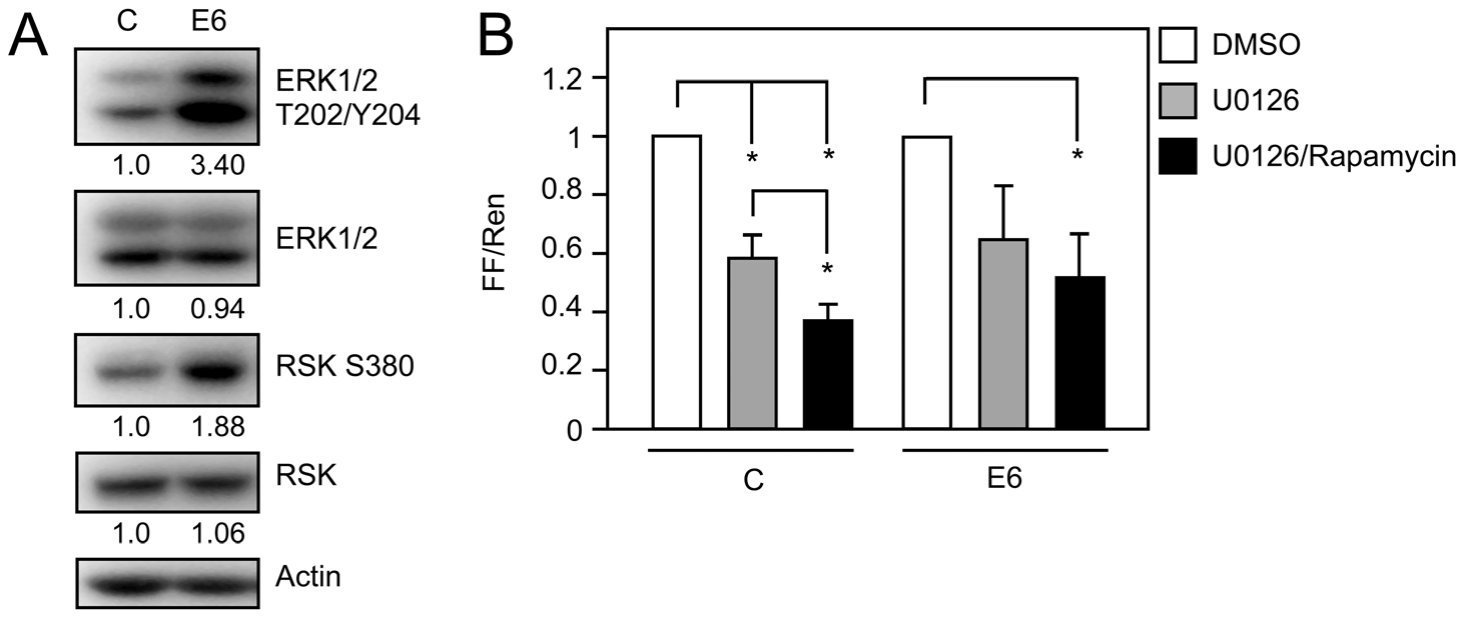
Signaling pathways downstream of RPTKs are activated in HPV16 E6 expressing HFKs. (**A**) Western blot analysis of MAPK signaling by analyzing ERK1/2 and RSK phosphorylation in HFKs with stable expression of HPV16 E6 (E6) or LSXN control vector (C) and experiencing nutrient withdrawal by PBS starvation for 30 minutes. Quantifications relative to actin are shown below each panel. A representative experiment from a total of four independent experiments is shown. (**B**) HPV16 E6 mediated increase in cap dependent translation is dependent on MEK and mTORC1 signaling. U2OS cells were transfected with pFR_CrPV_xb and the indicated plasmids and processed for firefly and Renilla luciferase activity at 48 hours post transfection. 20 hours prior to lysis, cells were treated with DMSO, 10 µM U0126 (a MEK inhibitor), or a combination of 10 µM U0126 and 100 nM Rapamycin (an mTORC1 inhibitor). Firefly and Renilla luciferase were normalized to levels in DMSO treated cells and are presented as fold changes of normalized firefly relative to normalized Renilla activity. The bar graph represents averages and standard deviations of three independent experiments, each performed in triplicate; the asterisk indicates statistical significance (*P*<0.05).

It has been reported that HPV16 E6 activates mTORC1 [16], [17], and that E6 mediated mTORC1 activation causes an increase in translation of capped mRNAs [16]. HPV16 E6 has also been reported to activate FAK signaling, which is also linked to RPTK activation [26], [27]. Given that we detected prolonged EGFR Y1068 and Y1173 phosphorylation, and INSRβ/IGFRβ phosphorylation at Y1150/51 and Y1135/36, respectively, which are associated with initiation of RAS/MAPK signaling, we evaluated MAPK activity under conditions of EGF withdrawal. We observed prolonged phosphorylation and activation of the MAPK effectors ERK1/2 in HPV16 E6 expressing HFKs, and the ERK substrate RSK was phosphorylated at higher levels in HPV16 E6 expressing HFKs than in control cells (Figure 2A). In addition to mTORC1, RAS/MAPK signaling can activate cap dependent translation through RSK mediated phosphorylation of the ribosomal protein S6 on serine residues 235 and 236 (S6 S235/36). To determine if MAPK signaling is required for HPV16 E6 to activate cap dependent translation, we utilized a bicistronic luciferase reporter assay [28]. U2OS human osteosarcoma cells were transiently transfected with the reporter construct and a vector expressing HPV16 E6 or empty vector, and MAPK activity was inhibited with the MEK inhibitor U0126. We found that cap dependent translation in control as well as E6 expressing cells is sensitive to MEK inhibition (Figure 2B, grey bars). We previously reported that HPV16 E6 activates cap dependent translation at least in part through mTORC1 [16]. To determine the relative contributions of MAPK and mTORC1 activation, we co-treated cells with Rapamycin and U0126 to simultaneously inhibit mTORC1 and MAPK. Similar to control cells, co-treatment of HPV16 E6 expressing cells with Rapamycin caused an additional decrease in cap-dependent translation (Figure 2B, black bars; *P*<0.05). Western blots demonstrate that U0126 treatment effectively inhibits MEK signaling (Figure S1).

### Reduced mTORC1 activity after EGFR or INSR/IGFR inhibition in HPV16 E6 expressing HFKs

**Figure 3:**
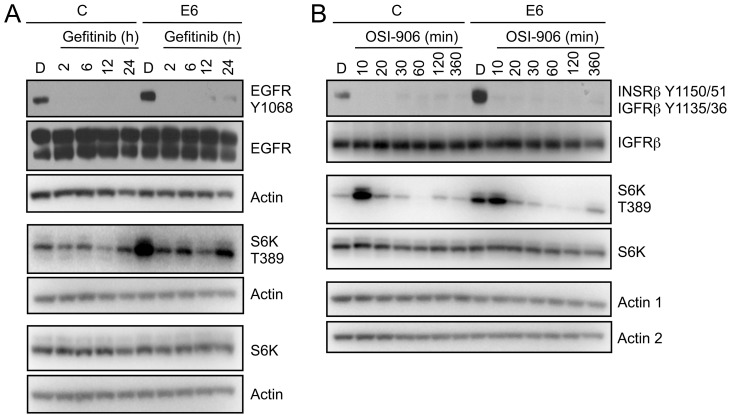
RPTK inhibition reduces mTORC1 activation in HPV16 E6 expressing HFKs. (**A**) Western blot analysis of EGFR and mTORC1 activation (S6K) in HFKs with stable expression of HPV16 E6 (E6) or pLentiN6.3 control vector (C) following EGFR inhibition with Gefitinib. Cells were treated with DMSO or 1 µM Gefitinib over a 24 hour time course. Actin blots are shown as loading controls. The experiment shown here represents one of three independent experiments. (**B**) Western blot analysis of INSRβ/IGFRβ and mTORC1 activation (S6K) in HFKs with stable expression of HPV16 E6 (E6) or pLentiN6.3 control vector (C) following INSRβ/IGFRβ chemical inhibition with OSI-906. These data represent one of two independent experiments. Cells were treated with DMSO or 150 nM OSI-906 over a 6 hour time course. Actin blots are shown as a loading control; “actin 1” corresponds to the blots with the phosphospecific antibodies, “actin 2” corresponds the other blots.

To determine whether RPTK activity is necessary for HPV16 E6 mediated mTORC1 activation, we treated HPV16 E6 expressing HFKs and control HFKs growing in normal culture media (KSFM) with the EGFR inhibitor Gefitinib and analyzed the phosphorylation status of the mTORC1 substrate S6 kinase (S6K). As predicted, we observed a rapid reduction of EGFR Y1068 autophosphorylation in control and HPV16 E6 expressing HFKs (Figure 3A). EGFR inhibition with Gefitinib treatment also partially inhibited S6K phosphorylation. Treatment with OSI-906, a dual kinase inhibitor of INSRβ and IGFRβ, caused rapid INSRβ and IGFRβ dephosphorylation in control and HPV16 E6 expressing HFKs (Figure 3B). At short time points after OSI-906 treatments, we reproducibly observed a spike of increased S6K phosphorylation in both control and HPV16 E6 expressing cells. This might represent compensatory S6K phosphorylation, perhaps via EGFR/PI3K/AKT signaling, consistent with a study that reported AKT activation upon MAPK inhibition [29].

These results suggest that EGFR and INSRβ/IGFRβ RPTKs each contribute to mTORC1 activation in HPV16 E6 expressing HFKs, under normal growth conditions.

### Increased internalization of activated receptor species in HPV16 E6 expressing HFKs

**Figure 4:**
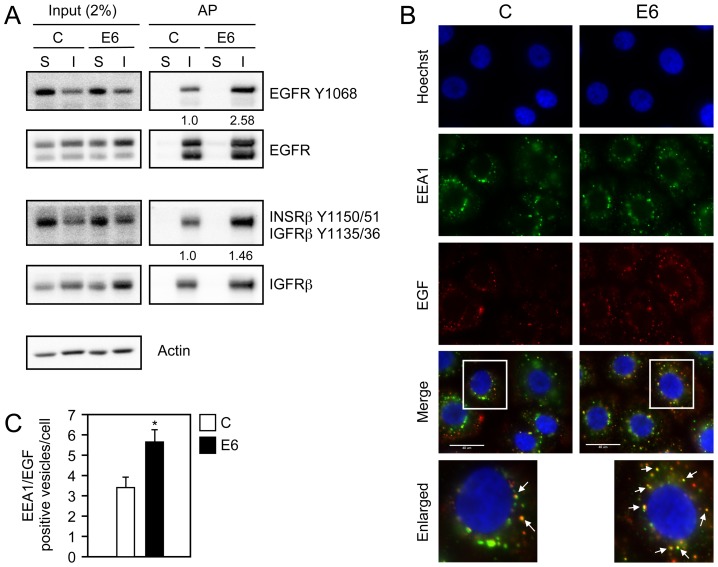
Internalization and degradation of activated receptor species are increased in HPV16 E6 expressing HFKs. (**A**) Western blot analysis of EGFR and INSRβ/IGFRβ following receptor internalization. After washing the cells with PBS they were incubated with biotin disulfide N-hydroxysulfosuccinimide ester for 40 minutes at 4°C followed by inactivation of the biotin reagent with NH_4_Cl for 10 min at 4°C. Cells were then stimulated with KSFM lacking growth factors and supplements for 3 hours at 37°C. Following ligand independent stimulation, cells were either lysed without reduction to measure surface bound receptors (S) or reduced and lysed to measure ligand independent internalization (I), Internalization assays were performed as described in the Materials and Methods. Levels of EGFR and INSRβ/IGFRβ in a 5 µg sample, representing 2% of the internalization assay, together with actin, are shown in the left panel (Input), and the resulting streptavidin affinity purification (AP) is shown in the right panel. Quantification representing the ratio of phosphorylated receptors to total receptors normalized to control vector (C) internalized phosphorylated receptors is shown below applicable panels. This experiment represents one of two independent experiments. (**B**) Immunofluorescence analysis of EGF co-staining with the endosomal marker EEA1 in HFKs with stable expression of HPV16 E6 (E6) or pLentiN6.3 control vector (C) following overnight starvation and stimulation with Alexa 647 labeled EGF (EGF) for 15 minutes. Cells were stained with Hoechst to visualize the nuclei. The scaling bar represents 40 µm. (**C**) Quantification of the number of EGF positive early endosomes from panel B. Bar graphs represent average numbers of EGF positive early endosomes per cell from four independent experiments. A total of 115 cells were evaluated. Error bars indicate standard deviations; the asterisk indicates statistical significance (*P* = 0.0017).

Since HPV16 E6 expressing cells exhibit prolonged RPTK and AKT activation after growth factor withdrawal, we evaluated internalization of activated EGFR, INSRβ and IGFRβ. HFKs were starved in PBS, and cell surface proteins were conjugated to biotin. The cells were then incubated in KSFM lacking EGF and other supplements for 180 minutes in order to promote ligand independent internalization. Cells were then treated with glutathione and lysed to measure internalized receptors. As a control, total cell surface receptors were measured by directly lysing cells without stimulation or subsequent glutathione reduction. Affinity purification with streptavidin conjugated beads was used to isolate biotin conjugated proteins and active receptor species were detected using phosphospecific antibodies. These experiments revealed that HPV16 E6 expressing cells show increased internalization of active, phosphorylated EGFR as well as INSRβ and/or IGFRβ in the absence of growth factors (Figure 4A).

To independently validate that HPV16 E6 increases EGFR internalization under different experimental conditions and by a different approach, we stimulated EGF-starved HFKs with Alexa-647 EGF and evaluated co-localization of EGF with the early endosomal marker autoantigen 1 (EEA1) after 15 minutes by fluorescence microscopy. Compared to matched vector transduced HFKs, HPV16 E6 expressing HKFs had an increased number of EGF positive early endosomes (Control = 3.40±0.54; HPV16 E6 = 5.67±0.65 EGF/EEA1 positive vesicles, *P* = 0.0017) (Figure 4B and C).

These results suggest that HPV16 E6 mediated activation of RPTK signaling and downstream effector cascades is correlated with increased internalization of activated receptors.

### The signaling adaptor protein GRB2 is critical for HPV16 E6 mediated EGFR internalization and mTORC1 activation

The signaling adapter protein GRB2 associates with EGFR phosphorylated at Y1068 and activates PI3K and MAPK signaling. In addition GRB2 has also been implicated as a critical mediator of EGFR internalization. GRB2 can interact with dynamin, a GTPase that is important for inclusion of receptors into vesicles during endocytosis [30].

**Figure 5:**
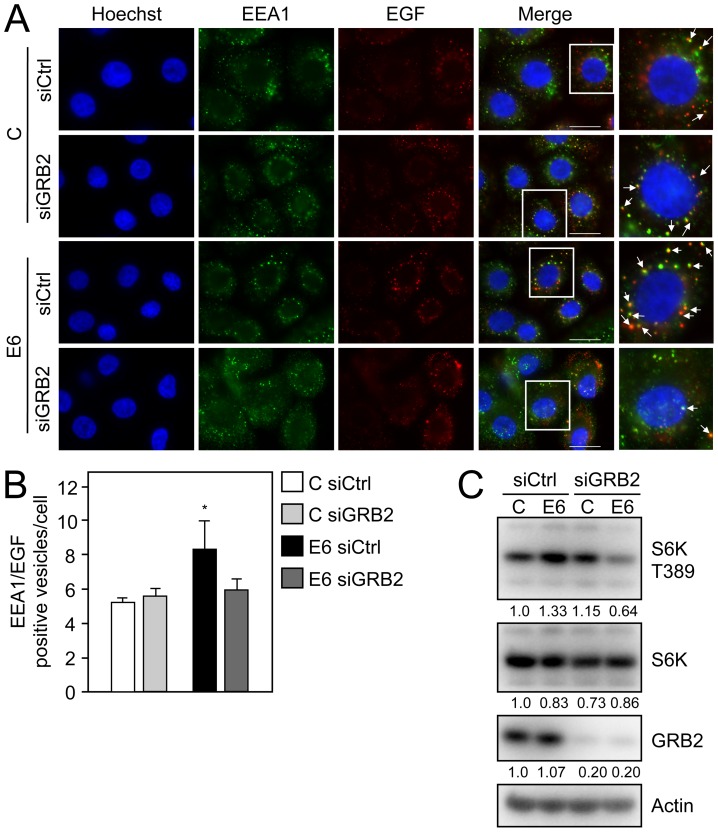
The signaling adaptor protein GRB2 is critical for HPV16 E6 mediated EGFR internalization and mTORC1 activation. (**A**) Immunofluorescence analysis of EGF co-staining with the endosomal marker EEA1 in GRB2 depleted (siGRB2) or control siRNA transfected (siCtrl) HFKs with stable expression of HPV16 E6 (E6) or pLentiN6.3 control vector (C) following overnight starvation and subsequent stimulation with Alexa 647 labeled EGF (EGF) for 15 minutes. Cells were stained with Hoechst to visualize the nuclei. The scaling bar represents 20 µm. (**B**) Quantification of the number of EGF positive early endosomes from panel A. Bar graphs represent average numbers of EGF positive early endosomes/cell with counts done in triplicate for a total of at least 95 analyzed cells per condition. Error bars indicate standard deviations; the asterisk indicates statistical significance (*P* = 0.03). (**C**) Western blot analysis of S6K T389 in GRB2 depleted (siGRB2) or control siRNA transfected (siCtrl) HFK populations with stable expression of HPV16 E6 (E6) or control vector (C). Cells were transfected with the corresponding siRNAs at 48 hours prior to lysis. An actin blot is shown as a loading control and quantifications relative to actin are shown below each panel. A representative experiment taken from four independent experiments is shown in panel C.

To determine if GRB2 may be important for HPV16 E6 mediated EGFR internalization we depleted GRB2 by RNA interference and evaluated co-localization of EGF with EEA1 after 15 minutes by fluorescence microscopy. Similar to Fig. 4B and C, HPV16 E6 expressing primary HFKs transfected with siCtrl had an increased number of EGF positive early endosomes in comparison to control HFKs (Control siCtrl = 5.29±0.23; HPV16 E6 siCtrl = 8.37±1.61 EGF/EEA1 positive vesicles/cell, *P* = 0.03) (Figure 5A and B). However, GRB2 depletion ablated the increase in the number of EGF/EEA1 positive vesicles in HPV16 E6 expressing HFKs to levels similar to control cells (Control siGRB2 = 5.65±0.45; HPV16 E6 siGRB2 = 5.96±0.59 EGF/EEA1 positive vesicles/cell) (Figure 5A and B). Interestingly, GRB2 depletion in control HFKs had no effect on the number of EGF positive EEA1 vesicles per cell.

We next utilized GRB2 depletion to determine whether GRB2 function is important for HPV16 E6 mediated mTORC1 activation. GRB2 depletion markedly reduced phosphorylation of the mTORC1 surrogate marker S6K in HPV16 E6 expressing HFKs. Interestingly, however, there was no detectable reduction of S6K phosphorylation when GRB2 was depleted in control HFKs (Figure 5C).

These results show that GRB2 is required to enhance EGFR internalization and likely perpetuate signal transduction originating from internalized receptors in HPV16 E6 expressing cells. Further, even though HPV16 E6 expression causes increased EGFR phosphorylation at multiple tyrosine residues and therefore is predicted to activate multiple downstream signaling pathways, signaling through GRB2 is critical for HPV16 E6 mediated mTORC1 activation.

### Increased cellular migration through a RPTK dependent mechanism in HPV16 E6 expressing HFKs

**Figure 6:**
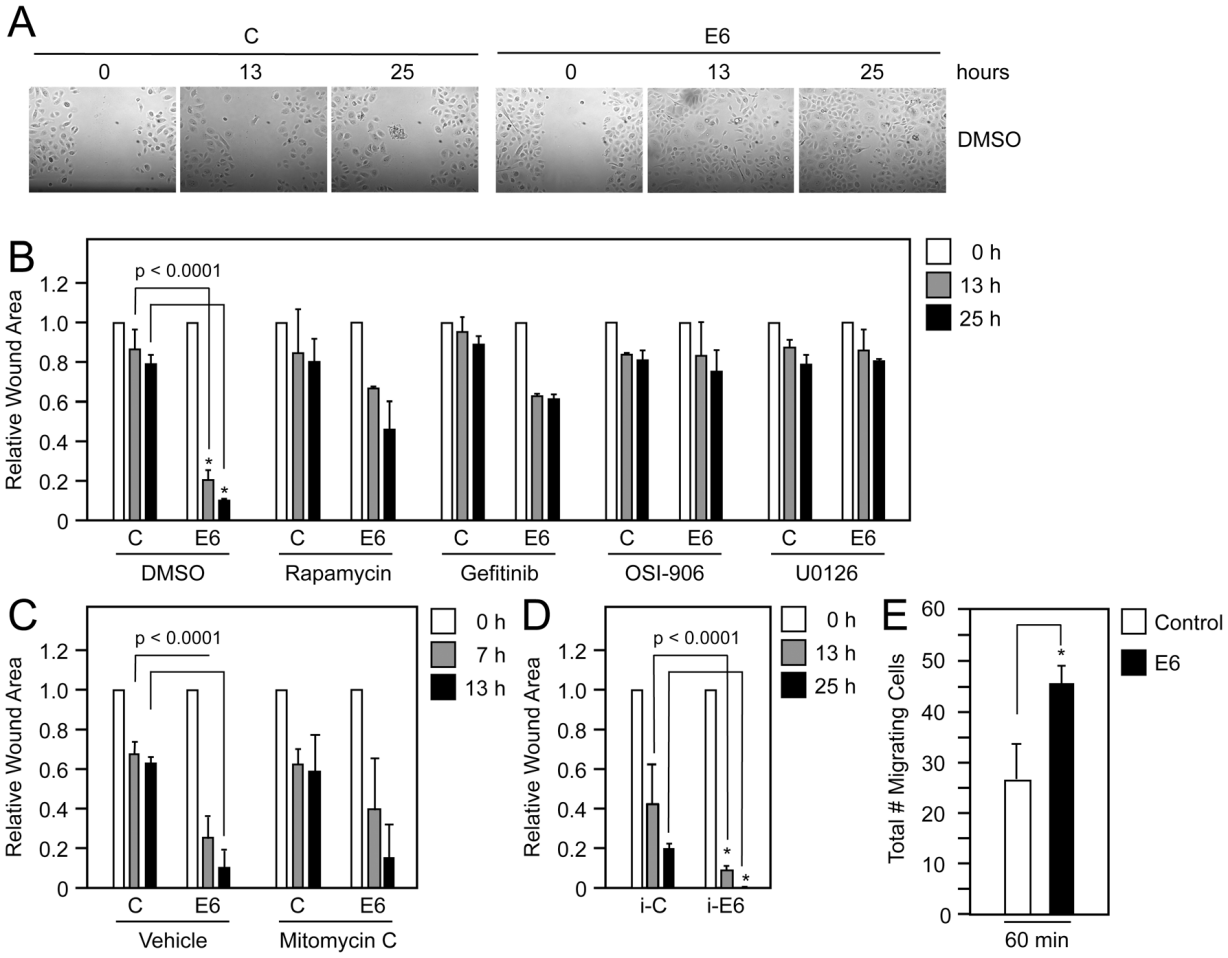
HPV16 E6 increases cellular migration in EGF depleted cells. (**A**) Wound healing assays with primary HFKs stably expressing HPV16 E6 (E6) or infected with pLentiN6.3 control vector (C) following wounding of the cellular monolayer in the absence of EGF following RPTK and effector pathway inhibition. Cells were treated with DMSO or 100 nM Rapamycin, 1 µM Gefitinib, 150 nM OSI-906, or 10 µM U0126 and closure of the monolayer was measured over a 25 hour time course. Only images of the DMSO treated samples are shown (**B**). Quantification of wound closure as shown in (**A**). Surface areas of wounds were calculated relative to the surface area of the wounds at t = 0 hour. Bar represent averages and standard deviations of three experiments; asterisks indicate statistical significance (*P*<0.0001). (**C**) Quantification of wound closure in the presence of 8 µg/ml Mitomycin C to inhibit cellular proliferation. (**D**) Quantification of wound closure in immortalized HFKs stably expressing HPV16 E6 (E6) or pLentiN6.3 control vector (C) (**E**). Transwell migration assay with HFKs stably expressing HPV16 E6 (E6) or pLentiN6.3 control vector (C). Cells were seeded in the absence of EGF on the top chamber of a transwell insert and migration to the bottom chamber in the absence of EGF was determined at 30 minutes and 60 minutes after seeding. The bars represent averages and standard deviations of three experiments; the asterisk indicates statistical significance (*P*<0.05).

We have shown that HPV16 E6 activates two RPTK effector signaling pathways: mTORC1 [16] and the MAPK pathway (Figure 2). Activation of these two pathways promotes cellular events that are associated with carcinogenesis including cellular migration (reviewed in [31], [32]). Therefore, we tested the hypothesis that HPV16 E6 mediated RPTK activation may increase cellular migration by performing wound healing and transwell migration assays. Wound healing assays were performed by scratching primary or hTERT immortalized HFK monolayer cultures grown in EGF deficient media and following wound closure over time. HFKs with stable HPV16 E6 expression migrated to close the wounded area in the absence of EGF with greater efficiency than control vector transduced HFKs (Figure 6A and B, relative reduction in the wound surface area at t = 13 h, 0.2±0.06 and 0.87±0.09, respectively; *P*<0.0001). We next determined if RPTK inhibition may inhibit cellular migration in HPV16 E6 expressing HFKs. Treatment with the EGFR inhibitor Gefitinib or the INSR/IGFR inhibitor OSI-906 impaired increased migration of HPV16 E6 expressing cells to a level similar to that of empty vector expressing cells (Figure 6A and B relative reduction in wound surface area at t = 13 h, 0.63±0.01 and 0.96±0.07; *P* = 0.86 (Gefitinib), 0.83±0.05 and 0.84±0.17 (OSI-906); *P* = 0.1103, respectively). As expected, inhibition of mTORC1 or MEK signaling similarly inhibited increased migration of HPV16 E6 expressing cells to levels of control HFKs (Figure 6A and B, relative reduction in surface area at t = 13 h, 0.67±0.01 and 0.85±0.22; *P* = 0.3716 (Rapamycin), 0.86±0.1 and 0.87±0.04; *P* = 0.9816 (U0126), respectively.) HPV16 E6 also increased cellular migration in wound healing assays in the presence of EGF and this was dependent on RPTK activity (Figure S2). To confirm that the ability of HPV16 E6 to promote wound closure was independent of cell proliferation, we inhibited proliferation with Mitomycin C and evaluated wound closure. As expected, HPV16 E6 expression enhanced cellular migration in the presence and absence of Mitomycin C (Figure 6C). Moreover, expression of HPV16 E6 in hTERT immortalized HFKs also caused increase cellular migration in wound healing assays (Figure 6D).

To independently validate these results, we also evaluated if HPV16 E6 increases migration of HFKs through a size restricting membrane in a transwell migration assay in the absence of EGF. These experiments could be performed over a much shorter time course than the wound healing assays, which further alleviates concerns regarding potential contributions of cell division. Enhanced migration of HPV16 E6 expressing cells through the membrane was observed as early as one hour in cells grown in the absence of EGF (Figure 6E, total number of migrating cells at t = 1 h, Control: 27±6.69; E6: 45.55±3.66; *P* = 0.04).

These results suggest that HPV16 E6 expression causes increased migration of primary HFKs in the absence of EGF, and that EGFR/INSR/IGFR effector signaling pathways are required for efficient HPV16 E6 mediated cellular migration.

## Discussion

We previously presented evidence that HPV16 E6 causes mTORC1 activation and increases cap dependent translation through PDK1 and mTORC2 dependent mechanisms [16]. While the mechanism of HPV16 E6 induced mTORC2 activation remains unknown, we focused on investigating signal transduction pathway that results in enhanced PDK1 activity in HPV16 E6 expressing HFKs. PDK1 phosphorylation is a result of increased RPTK and PI3K signaling, which is regulated by the activity of protein and lipid phosphatases. We evaluated the total protein levels of PTEN and several other phosphatases that act in this pathway and found no change in total protein levels in HPV16 E6 expressing HFKs in comparison to control cells under normal growth conditions or nutrient deprived conditions (Figure S3A, B). Since PTEN activity is regulated by subcellular localization [33], [34], we performed immunofluorescence analyses. There was no evidence for changes in PTEN subcellular localization in HPV16 E6 expressing HFKs as compared to control HFKs (Figure S3C). HPV16 E6 was previously shown to associate with the PTPN3/PTPH1 and PTPN13 tyrosine phosphatases and target them for proteasome mediated degradation [5], [10]. We did not detect an association of HPV16 E6 with PTPN3 or PTPN13, and therefore did not address whether and/or how these associations may contribute to HPV16 E6 mediated RPTK activation.

Here we present evidence that the PDK1 dependent component of mTORC1 stimulation is activated through RPTKs including ERBB2, EGFR, INSR and IGFR (Figure 1). Interestingly HPV16 E6 expression causes RPTK hyperactivation in the presence of growth factors and causes prolonged activity when ligands are withdrawn. Activation of downstream signaling cascades including the MAPK pathway was also demonstrated. EGFR or INSR/IGFR inhibition abrogated HPV16 E6 mediated mTORC1 activation as indicated by reduced phosphorylation of the surrogate marker S6K. MAPK inhibition by U0126 caused a decrease in cap dependent translation and combined inhibition of MEK and mTORC1 further reduces cap dependent translation. Hence HPV16 E6 activates cap dependent translation through two at least partially independent signaling pathways, mTORC1 and MAPK, that are both downstream of RPTKs. These results support numerous previous studies that have reported activation of signaling cascades downstream of RPTKs in HPV16 E6 expressing cells [5], [35]–[38]. For example, HPV16 E6 has been reported to activate FAK signaling, causing disruption of the cellular cytoskeletal structure [26], [27].

Even though HPV16 E6 and E7 have each been reported to transcriptionally activate EGFR expression [39], [40], our experiments did not provide evidence for increased EGFR or INSR/IGFR protein levels in HPV16 E6 expressing HFKs.

EGFR activation has also been studied in the benign respiratory papillomas that are caused by low-risk mucosal HPV6b and HPV11 infections and HPV6b and HPV11 E6 proteins have been linked to increased EGFR expression [41]. EGFR expression was not due to a gene amplification event, nor was it associated with an increase in mRNA expression. Instead, low-risk mucosal HPV E6 proteins have been reported to increase EGFR recycling to the cell surface by approximately 20%. The low-risk HPV E6 associated increase in EGFR expression was accompanied by enhanced EGFR tyrosine phosphorylation and increased MAPK activity [41]. In contrast to these studies, we did not detect an increase in total receptor levels in HPV16 E6 expressing HFKs, but rather a specific increase in phosphorylated, active receptor species.

We demonstrate that HPV16 E6 increases internalization of phosphorylated, active EGFR, INSRβ and IGFRβ in the absence of growth factors (Figure 4). Consistent with these studies we also report that HPV16 E6 expression enhances EGF localization, presumably bound to EGFR, to early endosomes, suggesting that HPV16 E6 activates RPTK signaling in the presence or absence of growth factors. RPTK activation causes increased receptor internalization, and internalized receptors maintain signaling potential since they are phosphorylated at residues that promote receptor association with signaling adaptor proteins and cause activation of downstream signaling cascades [24]. Moreover, EGFR receptor internalization is required for the maintenance of AKT activation [24], [42].

Our results show that GRB2 is an important mediator of the HPV16 E6-mediated increase in receptor internalization as GRB2 depletion reduces EGF internalization specifically in HPV E6 expressing cells (Figure 5). The molecular mechanisms accounting for this observation remain to be determined.

The mechanics of GRB2-receptor association and activation is different for different RPTKs. GRB2 associates directly with phosphotyrosine 1068 of activated EGFR [43]. In contrast, INSR and IGFR form indirect interactions with GRB2 via the IRS-1 and SHC adaptor proteins [44]. The PDGFR/GRB2 interaction is indirect, with SHP-2 mediating the association [45]. Similarly, the EPHA2-GRB2 association is also indirect, requiring SHC to form a complex that then leads to the activation of downstream signaling cascades including MAPK activation [46]. Given that GRB2 is an important effector of multiple RPTKs and that only a subset of these receptors are activated in HPV16 E6 expressing HFKs (Figure 1), E6 may only function through GRB2 bound to specific adaptors that are required for receptor internalization.

**Figure 7:**
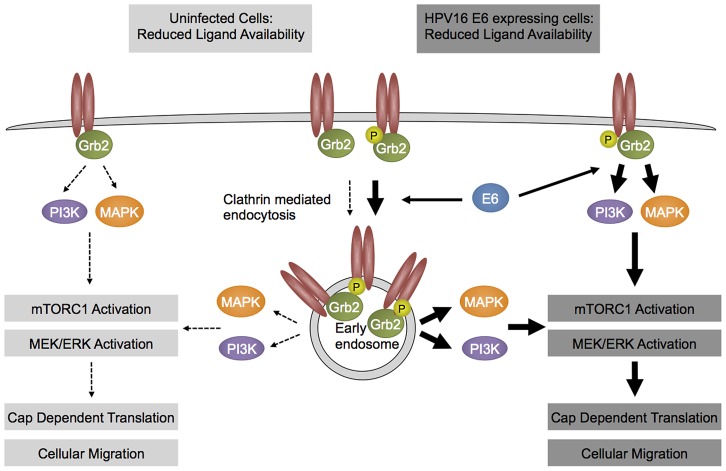
Hypothetical model. HPV16 E6 expressing cells (right) show enhanced activation of RPTKs at least in part by enhancing internalization of activated receptor species as compared to parental cell (left). As a consequence, downstream signaling circuits, including mTORC1 activity remain active even under conditions of limited growth factor availability. See text for details.

Taken together, these results form the basis for a model of how high-risk HPV E6 oncoproteins may contribute to enhanced receptor activation (Figure 7). We demonstrate that HPV16 E6 expression causes receptor hyperactivation under conditions of abundant growth factor availability, and that HPV16 E6 causes prolonged receptor activation under conditions of growth factor or nutrient deprivation. We postulate that the HPV16 E6-mediated RPTK phosphorylation recruits GRB2 to the cell membrane where it functions as a signaling adaptor for PI3K and MAPK effector pathways and participates in receptor internalization. According to this model, prolonged activation of the PI3K and MAPK effector signaling pathways enhances cap dependent translation and cellular migration.

E5 is the major transforming protein of the bovine papillomavirus type 1 (BPV1). This small, single pass transmembrane protein transforms cells by forcing dimer formation and thus activation of RPTKs including EGFR and PDGFR in the absence of any growth factor ligands [47]. It is tempting to speculate that HPV16 E6 and BPV1 E5 may have both evolved to short-circuit RPTK signaling for similar requirements of the viral life cycle, presumably to keep RPTK signaling active in virally infected cells so as to avoid elimination by cell intrinsic tumor suppressor mechanisms such as the trophic sentinel pathway [18]. However, BPV1 E5 is a much more potent oncogene as growth factor independent receptor dimerization will sustain RPTK activation indefinitely in the absence of growth factor since it forces dimerization and thus persistent activation of these receptors, whereas HPV16 E6 will only sustain signaling for a relatively short period of time after growth factor withdrawal.

The HPV E6 and HPV E7 proteins share biological activities with proteins encoded by other DNA tumor viruses, including polyomaviruses SV40, Merkel cell polyomavirus (MCPyV), murine polyomavirus, and adenoviruses. The PI3K/AKT/mTORC1 signaling axis is targeted for activation by many DNA tumor viruses. The ability of plasma membrane bound mouse polyomavirus Middle T antigen to activate AKT and other downstream mitogenic pathways through association and subsequent recruitment of the Class I PI3K p85 regulatory subunit has been well documented [48]–[51]. It was recently reported that the MCPyV small T antigen causes aberrant hyperphosphorylation and activation of eukaryotic translation initiation factor 4E binding protein (4E-BP1). This study claimed that 4E-BP1 activation is independent of mTORC1 or mTORC2, but no mechanism was proposed [52]. Hence, small DNA tumor viruses commonly activate PI3K/AKT/mTORC1 through multiple, diverse mechanisms. These mechanisms are not mutually exclusive and may account for activation of a variety of signaling pathways including MAPK, mTORC1 and FAK signaling.

Last but not least, these results also suggest that small molecule inhibitors of RPTKs and/or downstream effector pathways will deprive HPV16 infected cells of a crucial survival cue and may have therapeutic efficacy in HPV associated lesions and cancers.

## Materials and Methods

### Plasmids

Plasmids used in this study include the retroviral vectors pLXSN (control) and pLXSN HPV16 E6 [53]; and pCMV N (control) and pCMV HPV16 NE6no* [54]–[56]. Lentiviral vectors including pLentiN (control) and pLenti HPV16 NE6no* were generated by Gateway cloning into the pLenti6.3 Gateway compatible vector (Invitrogen). For the purposes of this study, the HPV16 E6 expression vectors were mutagenized such that they no longer have the capacity to generate the major splice variants [56], [57]. Mutagenesis yields a coding mutation in E6 (HPV16 E6 V42L) that does not interfere with the ability of HPV16 E6 to contribute to epithelial cell immortalization [57]. The pFR_CrPV_xb bicistronic firefly/Renilla luciferase vector [28] was obtained from Phil Sharp through Addgene (plasmid 11509) and was used to evaluate cap dependent translation as previously described [16], [56].

### Cell lines and culture

293T and U2OS cells (ATCC) were maintained in Dulbecco's modified Eagle medium (DMEM) (Invitrogen), supplemented with 10% fetal bovine serum (FBS), 50 U/ml penicillin and 50 µg/ml streptomycin. Primary human foreskin keratinocytes were isolated from anonymous newborn circumcisions as previously described [58], and maintained in keratinocyte serum-free medium (KSFM) supplemented with human recombinant epidermal growth factor 1–53, bovine pituitary extract (Invitrogen), 50 U/ml penicillin and 50 µg/ml streptomycin, 20 µg/ml gentamicin, and 1 µg/ml amphotericin B. Human hTERT immortalized cl398 keratinocytes (iHFKs) were obtained from Dr. A. Klingelhutz (U. Iowa). HPV oncogene expressing primary and immortalized HFKs were generated by retroviral infection with the corresponding pLXSN based vectors or by lentiviral infection with the corresponding pLenti6.3N based vectors following selection with 250 µg/ml G418 or 3 µg/ml blasticidin, respectively. All experiments performed with primary HFK populations were done so with donor and passage matched HFK populations passaged less than ten times. For nutrient deprivation assays, HFKs were grown to 90% confluence, at which point they were washed twice with phosphate buffered saline (PBS), followed by incubation in either PBS for 15 minutes or KSFM lacking EGF for 2 hours prior to analysis. HFKs were grown on poly-D-lysine coated plates (BD Biosciences) for PBS starvation experiments.

### Western blotting and antibodies

Unless otherwise indicated, protein lysates were prepared by incubating the cells in ML buffer (300 mM NaCl, 0.5% Nonidet P-40 [NP-40], 20 mM Tris-HCl [pH 8.0], 1 mM EDTA) supplemented with one complete EDTA-free protease inhibitor cocktail tablet (Roche) per 25 ml lysis buffer and one PhosSTOP phosphatase inhibitor cocktail tablet (Roche) per 7.5 ml lysis buffer on ice for 20 minutes. Cell lysates intended for global phosphotyrosine Western blots were prepared by incubating the cells in RIPA buffer (150 mM NaCl, 1% NP-40, 0.5% Deoxycholic acid [DOC], 0.1% SDS, 50 mM Tris-HCl [pH 7.5]), supplemented as described above for ML Buffer with the addition of 50 mM Pervanadate) on ice for 20 minutes. Cells were then scraped and lysates cleared by centrifugation at 16,110×g for 10 min at 4°C. Protein concentrations were determined using the Bradford method (Bio-Rad). Proteins were separated by SDS-PAGE and electrotransferred onto polyvinylidene difluoride membranes (Immobilon-P; Millipore). Unless otherwise noted, membranes were blocked in 5% nonfat dry milk in TBST (137 mM NaCl, 2.7 mM KCl, 25 mM Tris [pH 7.4], 0.1% Tween 20) and probed with the appropriate antibody. The following antibodies were used at a 1∶1000 dilution unless otherwise specified: β-Actin (1501; Chemicon), p53 (Ab-6, Calbiochem), GRB2 (ab86713, Abcam), Flag (4 µg/ml, F3165, Sigma), anti-phosphotyrosine (05-1050X, Millipore), EGFR (4267), EGFR Y992 (2235), EGFR Y1068 (3777), EGFR Y1173 (4407), S6K (9202), S6K T389 (9206), RSK (9333), RSK S380 (9335), ERK1/2 (9102), ERK1/2 T202/Y204 (4370), IGF-1Rβ (3027), IGF-1Rβ Y1135/36/INSRβ Y1150/51 (3024), PI3K p110α (4249), PI3K p110β (3011), PI3K Class III (3358), PTEN (9188), all from Cell Signaling Technology. Secondary anti-mouse and anti-rabbit antibodies conjugated to horseradish peroxidase were used at dilutions of 1∶10,000 or 1∶15,000, respectively. Proteins were visualized by enhanced chemiluminescence (Perkin Elmer, Millipore) and exposed on Kodak BioMax XAR film, or electronically acquired and quantified with a Kodak Image Station 4000R equipped with Kodak Imaging Software, version 4.0, or with a Carestream Gel Logic 4000 pro, equipped with Kodak Imaging Software, version 4.0.

### Receptor internalization assay

Primary HFKs were seeded into 15 cm dishes and internalization and degradation assays were performed when the cells reached 90% confluence. Cells were washed twice in ice-cold PBS-CM (PBS, 0.1 mM CaCl_2_, 1 mM MgCl_2_) followed by incubation in 5 ml of 0.5 mg/ml biotin disulfide N-hydroxysulfosuccinimide ester (sulfo NHS-SS-Biotin, Pierce) in PBS-CM by rocking at 4°C for 40 minutes. Cells were then washed two additional times in PBS-CM and incubated with PBS containing 50 mM NH_4_Cl for 10 min, rocking at 4°C, followed by two washes in PBS-CM. KSFM without any growth factors and supplements was added to one plate in order to investigate ligand independent receptor internalization over 180 minutes at 37°C. Following the 180 minute stimulation with KSFM lacking growth factors and supplements, cells were washed twice with PBS-CM and reduced by washing twice for 15 min each with Glutathione Buffer (50 mM reduced glutathione, 90 mM NaCl, 1 mM MgCl_2_, 0.1 mM CaCl_2_, 60 mM NaOH) rocking at 4°C. Cells were then washed twice with PBS-CM followed by one 15 min wash with PBS containing 50 mM Iodoacetamide (PBS-IAA), rocking at 4°C. Total cell surface receptors were measured by directly lysing one plate without stimulation or subsequent glutathione reduction. Lysates were then prepared by incubating cells with ID lysis buffer (200 mM NaCl, 75 mM Tris [pH 7.5], 2.5 mM EDTA, 2.5 mM EGTA, 1.5% Triton X-100, 0.75% NP40, 0.1% SDS), supplemented with one complete EDTA protease inhibitor cocktail tablet (Roche) and one PhosStop phosphatase inhibitor cocktail tablet (Roche). Receptor internalization assays were performed by pre-clearing lysate with 40 µl Pansorbin (Calbiochem) for 45 min, after which the Pansorbin was removed by centrifugation at 16,110×g for 10 min at 4°C. The pre-cleared lysate was then incubated with 40 µl of washed Streptavidin-agarose resin (Thermo Scientific) for 16 hours, rocking at 4°C, after which the beads were washed four times in ID buffer, 2× sample buffer was added, and proteins separated by SDS PAGE and electrotransferred onto PVDF for Western blotting.

### Immunofluorescence microscopy

For immunofluorescence analysis, cells were seeded onto glass coverslips 48 hours prior to fixation. Cells were washed two times with PBS and incubated with KSFM without any growth factors or supplements at 37°C for 16 hours. Cells were then stimulated with KSFM containing 150 µg/ml Alexa Fluor 647 EGF (Molecular Probes), fixed with 4% paraformaldehyde in PBS, washed with PBS, and permeabilized with 0.1% Triton-X100/1 mM Glycine in PBS for 10 min at room temperature. Following permeabilization, cells were washed with PBS and blocked first for one hour with 5% goat serum at 4°C and then with 2% BSA for 10 min at room temperature. Cells were incubated with anti-EEA1 (610457, BD) for one hour at room temperature. The secondary antibody was goat anti-mouse Alexa Fluor 488 (Molecular Probes) and was used at a 1∶1000 dilution. Nuclei were stained with Hoechst 33258. Images were acquired using a Nikon 80i upright microscope equipped with a Hamamatsu C8484-03 camera and processed using MetaMorph 7 software.

### Wound healing assay

Primary or immortalized HFKs were seeded into 6 cm dishes and a wound was introduced into the monolayer with a p200 pipette tip when the cells reached 90% confluence. Just prior to wounding the monolayer, the cells were washed two times with PBS and the media was replaced with KSFM lacking EGF for the minus EGF condition or with complete KSFM for the plus EGF condition, plus inhibitors when applicable. Cell proliferation was inhibited with the addition of 8 µg/ml Mitomycin C, where indicated. The cells were then incubated at 37°C and images of the wounded monolayer were captured over a time course using a Zeiss Axiovert 200 light microscope equipped with the Axiovision Release 4.4 SP2 software package. The wound surface area of each resulting image was calculated using Image J software (NIH). The surface area of the wound relative to the samples at t = 0 was determined. A total of at least 3 independent experiments were performed and statistical significance was calculated using the Student's T test.

### Transwell migration assay

Primary HFKs were trypsinized and resuspended in KSFM minus EGF. 30,000 HFKs were resuspended in 150 µl of KSFM minus EGF and placed in the upper chamber of a transwell permeable support membrane insert (8.0 µM, Costar product 3422) pre-wetted with 50 µl KSFM minus EGF. The bottom chamber was filled with 600 µl KSFM minus EGF, and the cells were incubated at 37°C for 30 minutes or one hour. Cells were then scraped from the upper chamber of the transwell membrane and the membrane was fixed with 100% methanol for 30 minutes and stained with Crystal Violet. Cells remaining on the underside of the membrane were then counted in three separate fields of view per experiment. A total of three independent experiments were performed and statistical significance calculated using the Student's T test.

### Transfections and luciferase assays

U2OS cells were transfected and processed for luciferase assays as described previously [16]. The fold change in activity was determined by calculating the ratio of firefly luciferase activity to Renilla luciferase activity compared to the control vector-transfected cells. Three independent experiments were performed and the Student's T test was used to calculate statistical significance.

### siRNA transfections

HFKs were seeded in 6 well dishes in triplicate and GRB2 siRNA (smart pool L-019220-00, Dharmacon) or scrambled siRNA was transfected with Lipofectamine 2000 (Invitrogen) to a final concentration of 40 nM. Lysates were prepared 72 hours post transfection.

## Supporting Information

Figure S1 **MEK inhibition reduces HPV16 E6 mediated activation of MAPK signaling.** Western blot analysis of MAPK signaling (ERK1/2 and RSK) in primary HFKs with stable expression of HPV16 E6 (E6) or LXSN control vector (C). Cells were treated with DMSO or 15 µM U0126 at 30 minutes prior to lysis.
(TIF)

Figure S2 **HPV16 E6 increases cellular migration in the presence of EGF.** **(A)**. Wound healing assay with HFKs stably expressing HPV16 E6 or pLentiN6.3 control vector following wounding of the cellular monolayer grown in “rich”, standard KSFM following RPTK and effector pathway inhibition. Cells were treated with DMSO or 100 nM Rapamycin, 1 µM Gefitinib, 150 nM OSI-906, or 10 µM U0126 and closure of the monolayer was measured over a 25 hour time course. **(B)**. Quantification of wound closure as shown in panel A. Surface area of wounds were calculated relative to the surface area of the wounds at t = 0 hour. The bars represent averages and standard deviations of four experiments for DMSO treated samples and two experiments for drug treated samples; asterisks indicate statistical significance (*P*<0.05).
(TIF)

Figure S3 **HPV16 E6 mediated activation of AKT/MAPK is not due to the destabilization or change in localization of PTEN and other phosphatases.** **(A)** Western blot analysis of the dual specificity phosphatase PTEN in HFKs with stable expression of HPV16 E6 (E6) or control vector (C) of under normal growth conditions (KSFM). **(B)** Western blot analysis of PTEN, SHP1/PTPN6 and PTP1b/PTPN1 in HFKs with stable expression of HPV16 E6 (E6) or control vector (C) under conditions of nutrient deprivation (PBS, 15 minutes). **(C)** Confocal immunofluorescence imaging of PTEN (green) subcellular localization in control or HPV16 E6 expressing HFKs. Nuclei are stained with DRAQ5 (blue).
(TIF)
