# Supplementary figures and images for: The HPV16 E6 Oncoprotein Causes Prolonged Receptor Protein Tyrosine Kinase Signaling and Enhances Internalization of Phosphorylated Receptor Species
Source: PLoS Pathog. 2013 Mar 14;9(3):e1003237. doi: 10.1371/journal.ppat.1003237 (PMC3597533; doi:10.1371/journal.ppat.1003237)

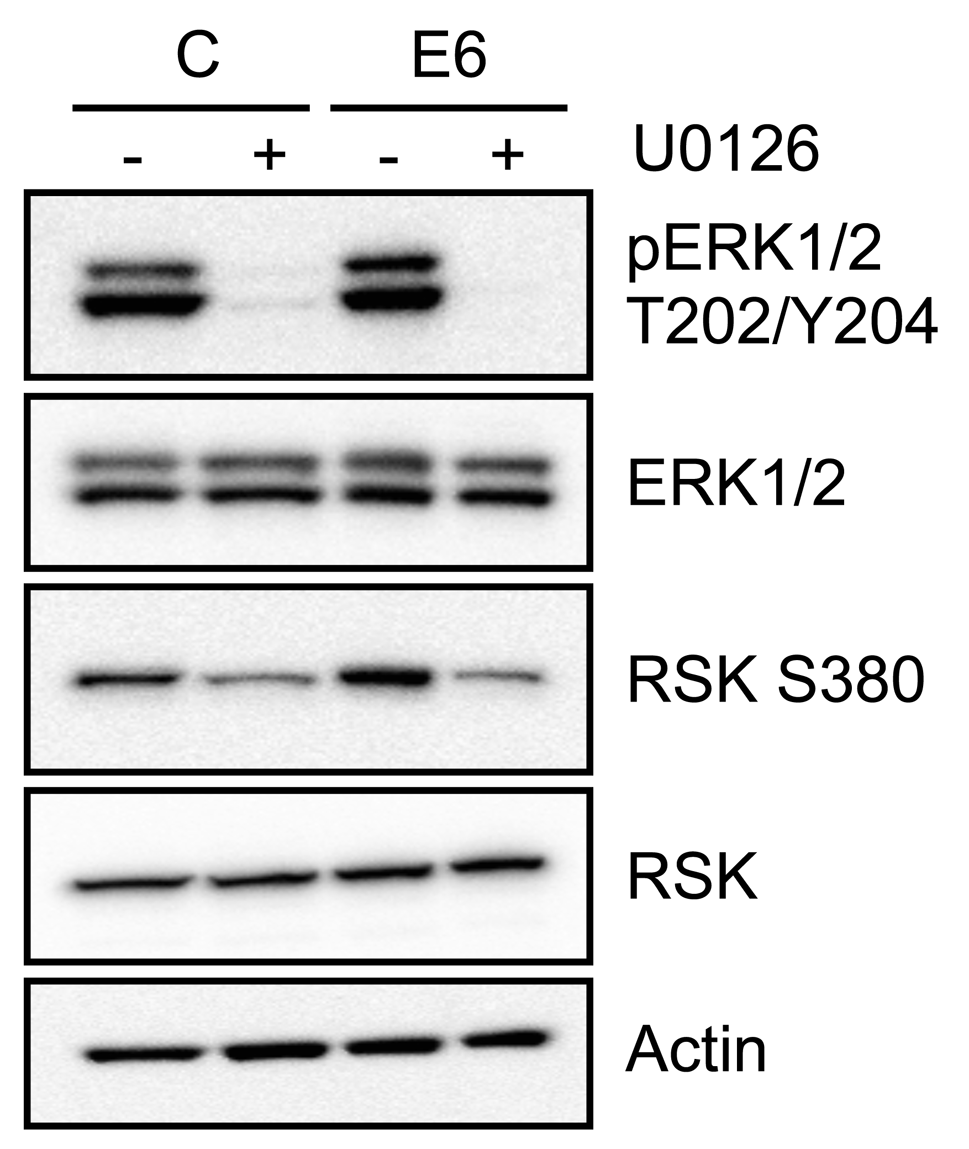

Supplement: Figure S1 — MEK inhibition reduces HPV16 E6 mediated activation of MAPK signaling. Western blot analysis of MAPK signaling (ERK1/2 and RSK) in primary HFKs with stable expression of HPV16 E6 (E6) or LXSN control vector (C). Cells were treated with DMSO or 15 µM U0126 at 30 minutes prior to lysis. (TIF) [file ppat.1003237.s001.tif]

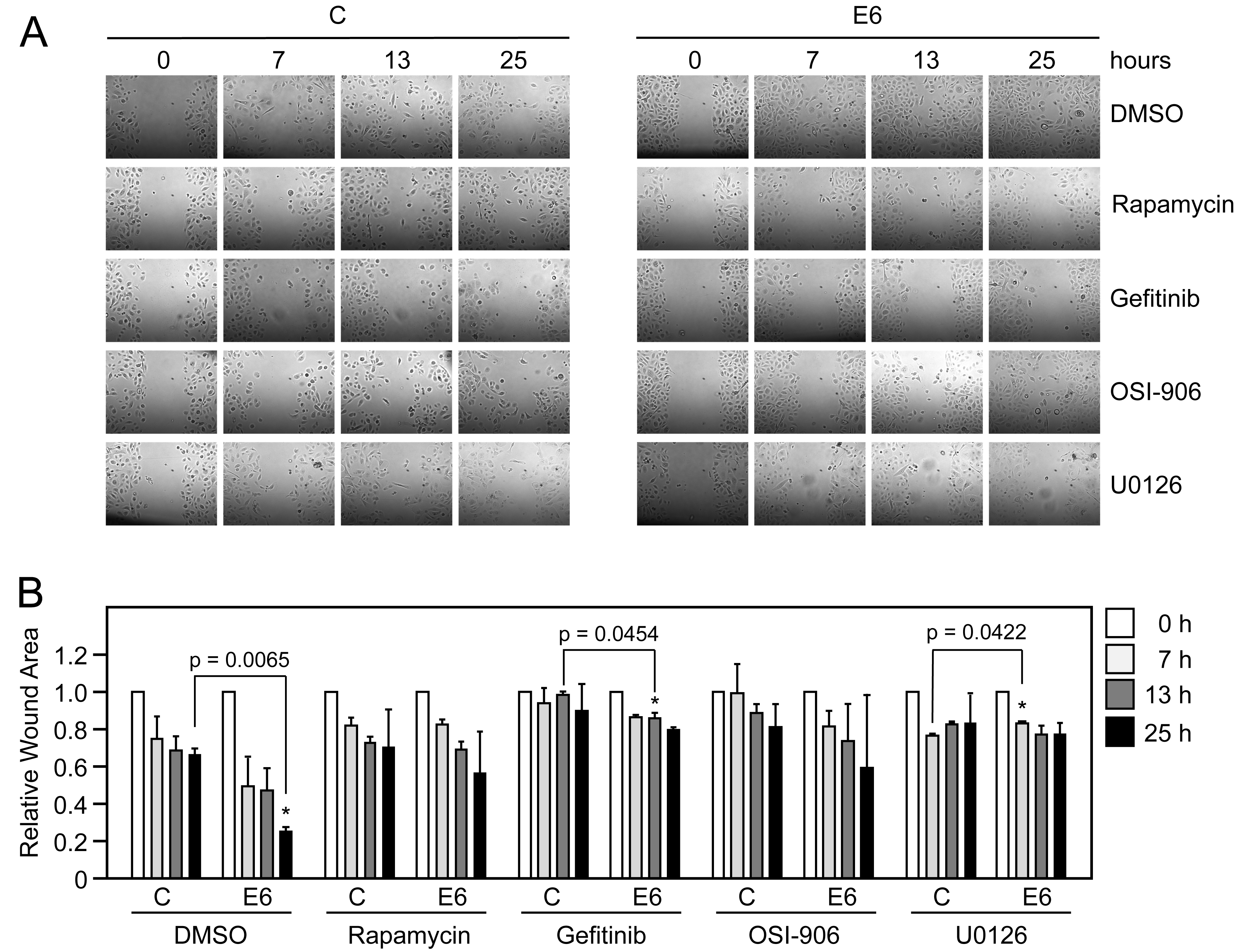

Supplement: Figure S2 — HPV16 E6 increases cellular migration in the presence of EGF. (A). Wound healing assay with HFKs stably expressing HPV16 E6 or pLentiN6.3 control vector following wounding of the cellular monolayer grown in “rich”, standard KSFM following RPTK and effector pathway inhibition. Cells were treated with DMSO or 100 nM Rapamycin, 1 µM Gefitinib, 150 nM OSI-906, or 10 µM U0126 and closure of the monolayer was measured over a 25 hour time course. (B). Quantification of wound closure as shown in panel A. Surface area of wounds were calculated relative to the surface area of the wounds at t = 0 hour. The bars represent averages and standard deviations of four experiments for DMSO treated samples and two experiments for drug treated samples; asterisks indicate statistical significance (P<0.05). (TIF) [file ppat.1003237.s002.tif]

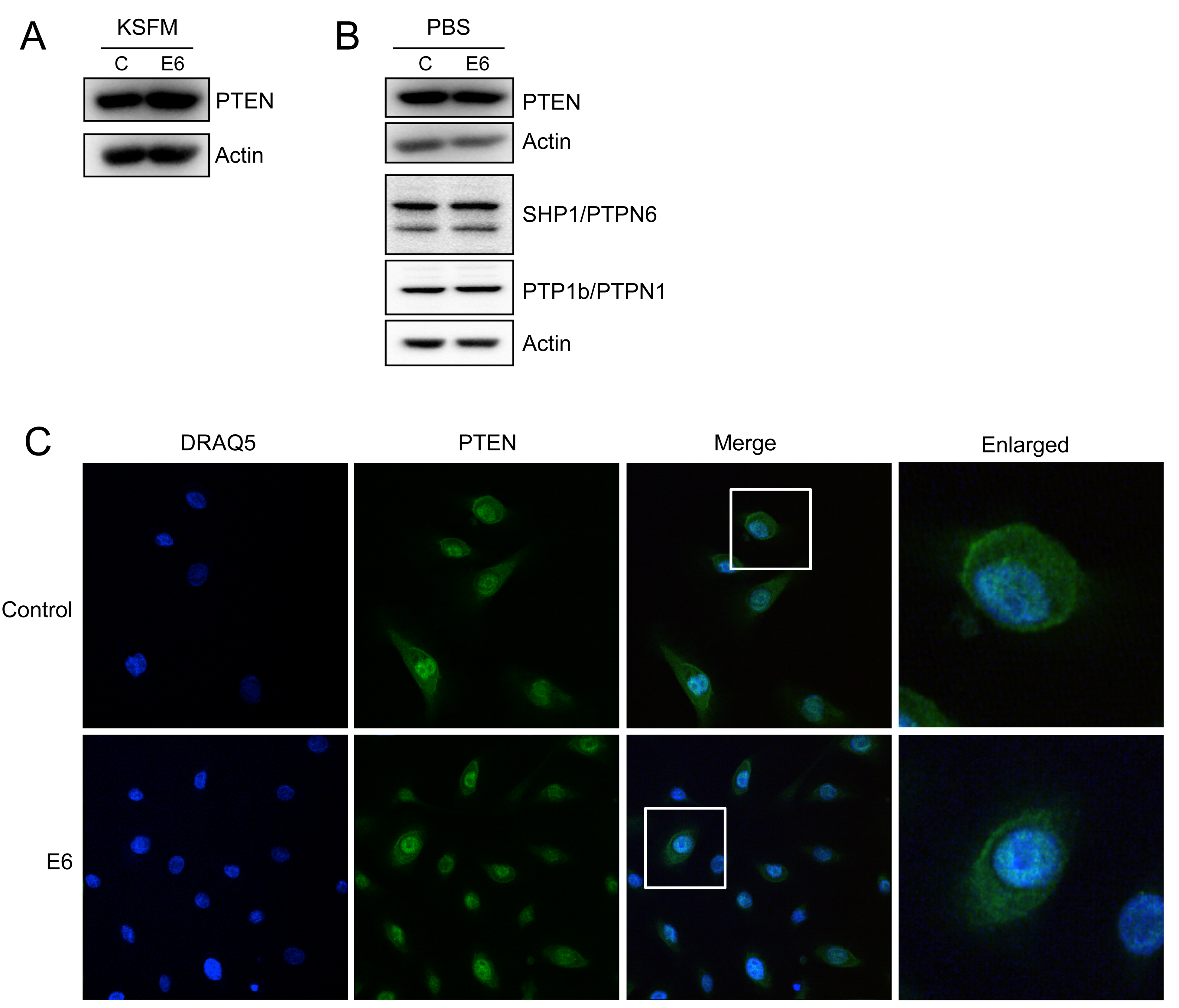

Supplement: Figure S3 — HPV16 E6 mediated activation of AKT/MAPK is not due to the destabilization or change in localization of PTEN and other phosphatases. (A) Western blot analysis of the dual specificity phosphatase PTEN in HFKs with stable expression of HPV16 E6 (E6) or control vector (C) of under normal growth conditions (KSFM). (B) Western blot analysis of PTEN, SHP1/PTPN6 and PTP1b/PTPN1 in HFKs with stable expression of HPV16 E6 (E6) or control vector (C) under conditions of nutrient deprivation (PBS, 15 minutes). (C) Confocal immunofluorescence imaging of PTEN (green) subcellular localization in control or HPV16 E6 expressing HFKs. Nuclei are stained with DRAQ5 (blue). (TIF) [file ppat.1003237.s003.tif]
